# How to decrease teenage pregnancy: rural perspectives in Ecuador

**DOI:** 10.3389/fpubh.2024.1370507

**Published:** 2024-05-01

**Authors:** Allpa Tituaña, Keren Herrán, Omar Galárraga, Iván Palacios

**Affiliations:** ^1^Colegio de Ciencias de la Salud - Escuela de Medicina, Universidad San Francisco de Quito, Quito, Ecuador; ^2^Arnold School of Public Health, University of South Carolina, Columbia, SC, United States; ^3^Department of Health Services, Policy and Practice, and International Health Institute, Brown University School of Public Health, Providence, RI, United States

**Keywords:** teenage pregnancy, Ecuador, rural population, indigenous population, community-based participatory research, evidence-informed intervention

## Abstract

**Introduction:**

This study aimed to understand the sociocultural context of teenage pregnancy in an Ecuadorian city with a large indigenous population, to gauge the acceptability of a multifaceted pregnancy prevention program for adolescents, and to elicit perspectives on the optimal program design from adolescents and adult key informants.

**Methods:**

We ascertained qualitative data via an online, electronic survey administered from August to September 2020. Open- and closed-ended questions elicited perspectives relating to burden of adolescent pregnancies, acceptability of pregnancy prevention programs, and optimal design of future programs. Twenty-four adolescents (13–19 years of age) and 15 adult key informants working in the healthcare, business, and education sectors in Cotacachi completed the survey. Survey responses were analyzed using a structural and *in vivo* coding, and an inductive approach to consensus-building around key themes.

**Results:**

Most adolescent survey respondents (75%) believed that teen pregnancy is “fairly common” in Cotacachi, and 41.7% believed differences in teen pregnancy rates are not associated with ethnicity. In comparison, 66.7% of adult survey respondents said teen pregnancy disproportionately occurs among indigenous teenagers. Additionally, 45.8% of adolescent and 80% of adult survey respondents believed that a comprehensive sexual education program would help reduce teenage pregnancy rates by imparting reliable sexual health knowledge. Adult respondents noted that the past programs were unsuccessful in preventing teenage pregnancy because of these programs’ inability to fully engage teenagers’ attention, very short time duration, or inappropriate consideration of cultural context.

**Discussion:**

In Cotacachi, Ecuador, a sexual health education program is both desired and feasible according to adult and teenager key informants. A successful program must adapt to the cultural context and engage youth participation and attention.

## 1 Introduction

Sexual and reproductive health “encompasses dimensions of physical, emotional, mental and social well-being in relation to sexuality” and it is not just the absence of disease (1). The lack of proper sexual and reproductive health services and education can contribute to the high incidence of teenage pregnancy in several countries (1–3). Adolescent mothers and their babies face very high risks for their health. In comparison to women 20–24 years old, adolescents have a higher risk of complications during pregnancy and childbirth such as eclampsia, postpartum endometritis, systemic infections (3) and ultimately, maternal mortality (4). Compared to neonates born to adult mothers, neonates born to adolescent mothers are more likely to experience low birth weight and other health complications derived from preterm delivery (3).

Latin America and the Caribbean (LAC) is one of the regions with the highest adolescent pregnancy rates in the world: there are 59 pregnancies for every 1,000 teenage women (15–19 years) (5). In comparison, Western Europe, Eastern Europe and Central Asia and North America have 8, 19 and 14 births per 1,000 adolescent girls and young women aged 15–19, respectively (6). In Ecuador, there are 64 pregnancies per every 1,000 adolescent women (5). Ecuador’s indigenous population represent 7.7% of the total country’s population (16′ 938.986) (7) and have historically been more vulnerable to adolescent pregnancy (8, 9). This vulnerability was exacerbated in 2014 when new restrictions to the Inter-Sectoral Strategy for Family Planning and Prevention of Adolescent Pregnancy program restricted reproductive health services for adolescent women, leading to increased birth rates by 8.5 births per 1,000 women in cantons with higher indigenous concentration (2).

Adolescent pregnancy also has severe socioeconomic effects. In Ecuador, the cost of caring for unintended pregnancy is over 5 times the cost of preventing it (10). A study among 6,487 young females (ages 15–19) in Ecuador estimated that school dropouts due to adolescent pregnancy represented a loss of $317 million in future income (10). Despite the health and economic impacts of unplanned adolescent pregnancies, funding for sexual and reproductive adolescent health is constantly threatened; for instance, the country’s teenage pregnancy prevention funds diminished from $5 USD million in 2019 to $0 USD in 2020 (11).

Despite evidence-based recommendations to include comprehensive sexuality education (CSE) as part of education curriculums several years ago (12), Ecuador is still working to implement it into the education system. In fact, the Ministry of Education approved in October 2023, the “National Strategy for Comprehensive Sexuality Education,” which will be implemented progressively in the education system until 2023 (13). The document is not publicly accessible; therefore, its content is still unknown. Previously to this announcement, educational authorities claimed that CSE it’s part of the education system following a “transversal” strategy (14), whereby every subject (math, science, physical education, etc) at some point addresses a sexual education topic. However, Ecuadorian students from both private and public schools reported that their schools did not offer any continual sexual health education and that they lacked knowledgeable and reliable sources of accurate sexual health information (15).

Comprehensive sexual education works best to decrease adolescent pregnancy when combined with economic empowerment (16). Economic empowerment increases decision-making power in relationships, and access to contraceptives (16). For example, a multifaceted program in Uganda that offered vocational training alongside sexual health education found that, 4 years post-intervention, adolescents receiving the program were 4.9 percentage points more likely to engage in income generating activities, and with sharp declines showed decreases in adolescent pregnancy, marriage, cohabitation, and rape (17). Also, teenagers’ beliefs regarding ideal ages at which to marry and bear children adjusted to an older age (17). A study of the Haiti Adolescent Girls’ Initiative found that providing technical training with socio-emotional and soft-skills development increased empowerment on key socioeconomic dimensions, autonomy in personal mobility, and self-assertion in the intimate circle (18).

Despite these promising results, there are also contradictory reports in the literature. For instance, the 2009 Liberian Economic Empowerment of Adolescent Girls and Young Women (EPAG) program provided technical, cognitive, and emotional skills training, which included education on sexual and reproductive health. Six months after this intervention, researchers found that there were no changes in sexual behaviors among participants. Researchers claimed that sexual behavior was not an objective of the program, therefore these results are explained due to the lack of specificity and longer-term of the intervention (19). This suggests that the efficacy and optimal design of multifaceted adolescent pregnancy prevention programs merits further investigation.

To our knowledge, there has not been a multifaceted teenage pregnancy intervention with indigenous population. Due to the social complexity, interventions like the multifaceted programs in Uganda and Haiti have the potential to work in LAC countries if properly adapted. Public health interventions should never be imposed upon a population (20). Instead, they should be culturally adapted to the needs and desires of the specific population the intervention is meant to benefit (20). Without proper cultural adaptation, the intervention may also prove wholly ineffective when applied to the context of another community.

The objective of this study is to understand the sociocultural context of teen pregnancy in a small city in Ecuador (Cotacachi) which has a high indigenous population. This study also aims to gauge the acceptability of a multifaceted teenage pregnancy prevention program and how one may be best designed according to teen and adult key informants within the community.

## 2 Methods

### 2.1 Study setting

Participant enrollment and data collection occurred in Cotacachi, Ecuador. Cotacachi is a city of approximately 53,001 inhabitants located in the north of the country (7). The city’s population is 80.1% rural and 41.7% of indigenous descent (7). In 2020, Cotacachi’s public health system reported 210 pregnancies among adolescents between 10 and 19 years of age (21).

### 2.2 Study population

The study population included adult key informants working in Cotacachi and adolescent males and females living in Cotacachi. Adults were recruited using a convenience sampling approach, selecting adults with experience working in the healthcare and education sectors who had experience working directly with adolescents or on topics of adolescent sexual health. We also recruited adults from the business sector with experience related to youth economic empowerment. We recruited a total of *n* = 15 adults to complete the survey, 5 from each of the three sectors. We additionally recruited *n* = 24 adolescents, ages 13–19, to complete the survey. The sample size was determined by data saturation. We consider data saturation when we identified redundancy in the data (22). Researchers were constantly monitoring the data collected and found data saturation with those participants.

Sexual health education is still taboo in Ecuador, especially in rural communities. Therefore, heterogeneous responses were appropriate to answer our research question, since the hypothetical program needs to be acceptable to youth, and also feasible and sustainable for adults to implement. This practice also agrees with data source triangulation, since it “involves the collection of data from different types of people, including individuals, groups, families, and communities, to gain multiple perspectives and validation of data.” (23).

The local investigator who resides in Cotacachi and worked at the Cotacachi Public Health Center contacted participants (adults and teenagers) via phone call or text message from contacts provided by people in the community. Eligible adults employed within the healthcare sector were recruited in person. All individuals provided voluntary online informed consent/assent for study participation, which was approved by the IRB. The institution granted an exempt condition to this study because it was a “Research with data collection in an anonymized and digital manner, without human contact” All individuals were required to have a Level-1 reading ability (corresponding to completion of the seventh year of basic education in the Ecuadorian educational system).

### 2.3 Survey design

We decided to conduct this study through an online survey due to the pandemic conditions. Despite this method is underutilize, it has been described that “qualitative surveys may be more appropriate when: they are the best ‘fit’ for participants’ needs (e.g., for very sensitive topics); a population is dispersed, hard to engage or access and/or diverse; a wide range of perspectives or positionings is sought […]” and that “*qualitative* survey datasets can provide richness and depth, when viewed in their entirety, even if individual responses might themselves be brief.” (24). We developed two surveys based on successful multifaceted programs components and the target audience: adolescents and adult key informants. Surveys were designed by the investigative team and reviewed by native Spanish speakers from the community to ensure cultural accuracy. Both surveys ascertained demographic information followed by 18 multiple-choice and open-response questions that asked about views on adolescent pregnancy and previous adolescent pregnancy prevention programs, the acceptability of a theoretical multifaceted teenage pregnancy prevention program, and suggestions for program design components. The adult survey additionally asked whether the respondent had adolescent children, and their place of residence and employment sector. All surveys were developed in Spanish and programmed using Google Forms. Each survey took between 20 and 30 min to complete.

### 2.4 Data collection and analysis

Following the Handbook of mixed methods in social behavioral research, our design uses qualitative and quantitative approaches in the types of questions, data analysis procedures, and inferences (25), and thus is considered mixed-methods research.

Recruitment and data collection occurred from August 17th to September 18th, 2020. At enrollment and immediately after providing informed consent, participants received the questionnaire via email or text message. Interviewees completed the survey in Google Forms on their personal computer or Smartphone. All responses were recorded anonymously, and participants could skip any question they did not want to answer.

The data collected in the Google form platform was downloaded by the local investigator and stripped of identifiers for analyses. First, the data were classified into two groups: adult key informants and adolescents. Second, the frequency (%) was obtained for all socio-demographic characteristics, dichotomous (yes/no) questions, and multiple-choice questions. Last, the research team analyzed answers to open questions using structural coding and *In vivo* coding, which uses participants’ own words to create an analytic coding scheme (26). Analysis was made in Microsoft Excel software (27). One of the investigators synthesized the major themes from each open question using an inductive approach. Meanings from these codes were used to produce clusters of themes, which were then discussed and agreed upon by the coding team over 5 iterations of reviews to create consensus (28). The research protocol was reviewed and approved by the bioethics committee of the Universidad San Francisco de Quito (Comité de Ética de Investigación en Seres Humanos, Study Code: 2020-036 M).

## 3 Results

### 3.1 Participants description

A total of *n* = 24 teenagers (13–19 years old) participated (Table 1). All of them lived in Cotacachi; three of them reported having a child at the time of the study. A total of *n* = 15 adult key informants (20–60 years old) participated in the study, five per each sector: education, healthcare, and commerce. Twelve of them lived in Cotacachi; nine of them reported having one or more children at the time of the study.

**Table 1:** Demographics of study participants.

Demographic categories	Teenagers *n* = 24		Key informants *n* = 15	
	N°	% of sample	N°	% of sample
Age (years)	13–15 y/o = 10 16–17 y/o = 8 18–19 y/o = 6	41.7% 33.3% 25%	20–40 y/o = 8 41–60 y/o = 7	53.3% 46.7%
Sex Female Male	11 13	45.8% 54.2%	9 6	60% 40%
Ethnicity (Self-identified) Indigenous Mestizo White	7 16 1	29.2% 66.7% 4.2%	3 11 1	20% 73.3% 6.7%
Education Middle school High school Professional degree	7 17 0	29.1% 70.8% 0%	0 1 14	0% 6.7% 93.3%
Marital status Single In a dating relationship Married Common law Divorced	19 3 0 2 0	79.2% 12.5% 0% 8.3% 0%	5 0 7 1 2	20.8% 0% 29.2% 4.2% 8.3%
Internet and technology access Internet at home and access to a computer Internet access with a smartphone only Internet at home and owns a tablet No internet at home, but owns a tablet	18 3 2 1	75% 12.5% 8.3% 4.2%	15	100%

Common law is a status in Ecuador when a couple live together and have children but are not legally married.

**Figure 1:**
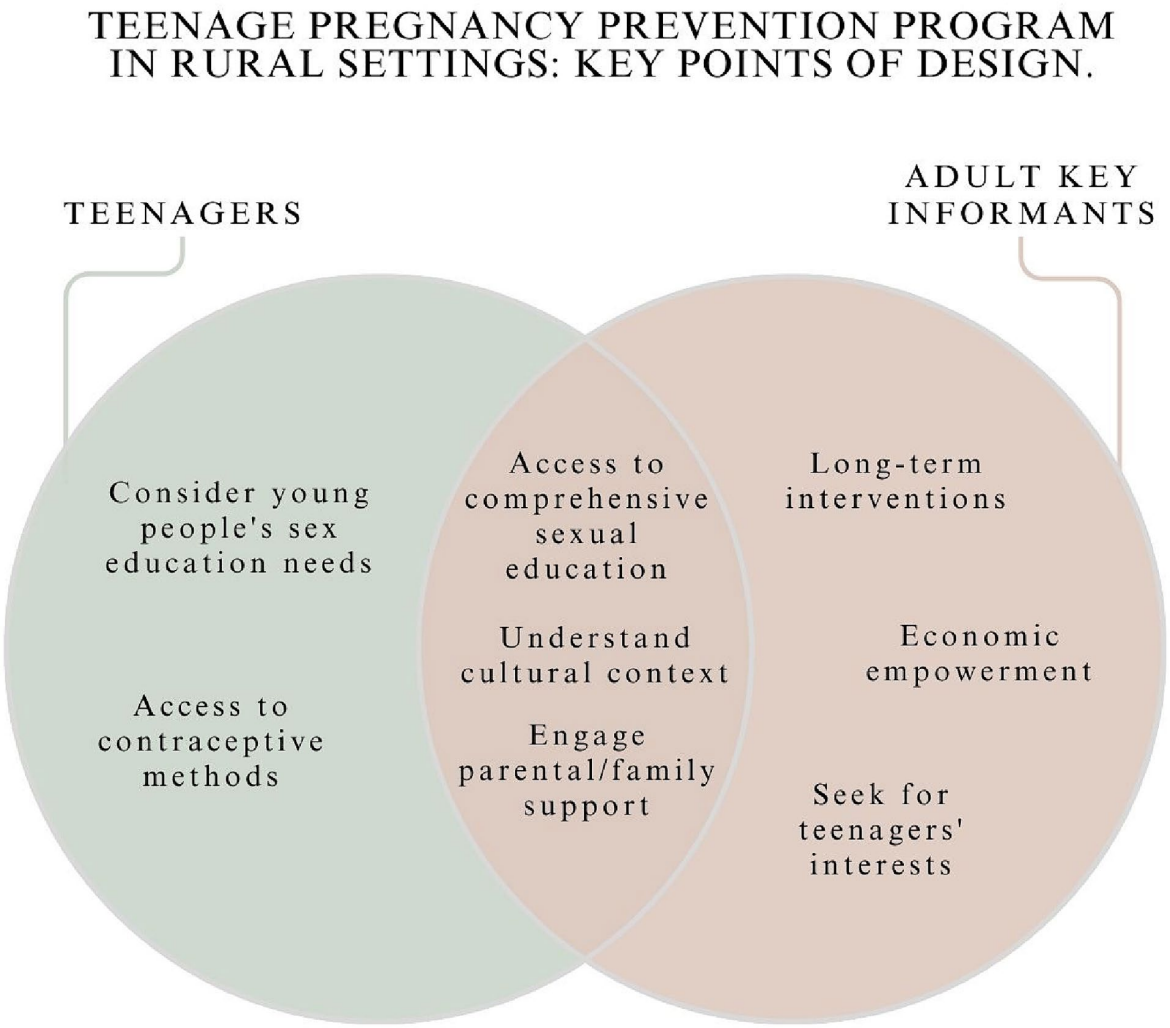
Key points to design a teenage pregnancy prevention program in a rural setting.

We wanted to understand the perceptions than teenagers and adults have regarding pregnancy during adolescence, and about prevention programs. Therefore, we divided the results in two main sections to helps us (1) understand the sociocultural context of teenage pregnancy in a city with high indigenous populations; and (2) gauge the acceptability of a multifaceted teenage pregnancy prevention program. By assessing this, we also have information about what are teenagers and adult key informants’ expectations about an intervention that seeks to prevent pregnancies during adolescence, so we can understand how one may be best designed in the sociocultural context. We contrast adult key informants’ interests with teenagers’ points of view. A summary of the key points that emerged from this study to design a teenage pregnancy prevention program in an Ecuadorian indigenous and rural population is provided at the end of this section (Figure 1).

### 3.2 Sociocultural context of teenage pregnancy

We explored teenage and adult key informants’ perceptions about teenage pregnancy. Qualitative results are described in Table 2. We present the most relevant findings in this section. Quantitative findings are described to provide as much detailed description about the study population and their preferences as possible.

**Table 2:** Themes about perceptions on adolescent pregnancy and an adolescent pregnancy prevention program.

Participant’s answer	Respondent(s)	Emergent theme(s)
Question: do you think adolescent pregnancy is common in Cotacachi? Why or why not?		
Adolescent pregnancy is very common or fairly common in Cotacachi	Adolescents	Lack of comprehensive sexual health education Little parental / family support Lack of use of contraceptive methods Sexual violence Irresponsibility Early initiation of sexual activity
	Adult key informants	Lack of comprehensive sexual health education Little parental / family support Culture and traditions Sexuality still treated as taboo Religious influence Irresponsibility Lack of use of contraceptive methods Family or sexual violence Greater risk in rural areas Immaturity Family or sexual violence Gender inequality Low economic level Early marriage
Question: Is any racial/ethnic group more vulnerable to adolescent pregnancy? If yes, which one and why?		
Indigenous are more vulnerable to pregnancy during adolescence	Adolescents	Their culture is different. Young parenting is normalized. Cultural ideology
	Adult key informants	Cultural customs Poverty Lack of access to education Machismo
There is not an ethnic group more vulnerable to adolescent pregnancy	Adolescents	Responsibility lies on each individual, not on their culture. An ethnicity does not conditionate an adolescent pregnancy. The risk of teenage pregnancy is the same across every ethnicity in Cotacachi.
	Adult key informants	Adolescent pregnancy occurs in every ethnicity. Ethnicity is not the only problem.
Question: Do you think that a program that combines economic empowerment with sexual health education could decrease the rate of adolescent pregnancy in Cotacachi? Please explain your answer.		
Yes	Adolescents	There is a need to receive sexual education. More education decreases vulnerability. Information can raise awareness about adolescent pregnancy consequences. Information can prevent from early initiation of sexual activity.
	Adult key informants	Economic empowerment can increase interest to educate in other fields. Economic empowerment and sexual education can lead to have other vision of life and life goals. Economic empowerment leads to women empowerment and women’s economic independence. Education improves decision-making capacity. This program could increase motivation to be an entrepreneurship.
Maybe	Adolescents	Not everyone could participate in the program. It depends on every individual interest. Some people do not take it seriously. It depends more on the local culture.
	Adult key informants	It depends on every individual interest. In most cases adolescents ignore these workshops.
No and I do not know	Adolescents	Irresponsible conduct would not change. It would increase the interest to experiment [sexual activities] Results depend on target audience and investigators effort.

#### 3.2.1 Do you think adolescent pregnancy is common in Cotacachi? Why or why not?

Out of the 24 adolescents that participated, 20.8% choose the option “Yes, it’s very common,” 75% choose “Fairly common” and 4.2% choose “No, it’s not common.”

Table 2 summarizes the themes that emerged. *Lack of comprehensive sexual health education* was the most mentioned reason by teenagers that believe that adolescent pregnancy was very common or fairly common. One participant noted, for example:

*“There is no accurate sexual health education, and we lack support and understanding from our parents when it comes to having a romantic partner.”*

Participant #19

Other reasons that the majority of adolescents claimed were *Little parental/family support* and *Lack of use of contraceptive methods.*

“*Lack of information and communication with parents, not using contraceptive methods or misusing them.”*

Participant #17

“Lack of trust with parents to talk about sexuality.”

Participant #7

Out of the 15 adult key informants, 66.7% choose the option “Yes, it’s very common” and 33.3% choose “Fairly common.” Similarly to adolescents, this group also identified lack of comprehensive sexual health education as the main cause of adolescent pregnancy in Cotacachi. They also agreed with teenagers regarding how, irresponsibility, family or sexual violence, and lack of access to contraception perpetuates teenage pregnancy. However, the reasons more mentioned were little parental/family support, sexuality still treated as taboo and, culture and traditions.

Most adult respondents gave multiple answers. For example, one participant stated:

“*[Teenage pregnancy is the result of] lacking access to preventative health information, sexual education by the educational system and in the family environment is deficient. Unfortunately, sexuality is still seen as a taboo; dogmas prevail in many cases.*”

Participant #4

#### 3.2.2 Is Any racial/ethnic group more vulnerable to teenage pregnancy? If yes, which one and why?

A third of adolescents (33.3%) think that indigenous are more vulnerable to get pregnant during adolescence. Out of those who answered, “indigenous people,” only three gave a reason, and stated that this happens because of their culture (Table 2).

“*The ethnic group that I think [is more vulnerable] is indigenous because there are many women and men who fall in love young and do not mind have children.”*

Participant #32

In contrast, 41.7% of adolescents think that there is not an ethnic group more vulnerable to teenage pregnancy. Similarly, only three of them gave a reason.

*“No, I think that everyone is responsible for what they do, and we should not necessarily be of an ethnic group to be more likely to have a young pregnancy.”*

Participant #26

Additionally, 4.2% of adolescents stated that both indigenous and mestizos^1^ are vulnerable to adolescent pregnancy; and 20.8% skipped this question.

Among adult key informants, 66.7% believed that indigenous ethnicity made teens in Cotacachi to be more vulnerable to early pregnancy. Only 3 could explain their reasoning (Table 2).

*“I think that the groups vulnerable to teenage pregnancy [are] mainly impoverished groups, where access to education is not essential, but rather survival. With these characteristics, indigenous groups may be more vulnerable.”*

Participant #13

Furthermore, 20% of adult key informants stated that there is not an ethnic group more vulnerable to teenage pregnancy, but they could not provide detailed explanations; and 13.3% skipped this question.

### 3.3 Multifaceted teenage pregnancy prevention program acceptability and design

#### 3.3.1 Previous teenage pregnancy prevention programs

We explored if there have been any teen pregnancy prevention programs in Cotacachi. Results are in Table 3. Teenagers could not specify any type of prior program and have contradictory opinions on their effectiveness. One claimed that prior programs had reduced teenage pregnancy, while another said that it was completely ineffective. The rest did not provide more information. This trend was also true for the adult key informants’ group. Out of the adults with knowledge about previous interventions, two said that the programs were part of the Ministry of Health, while the rest did not provide more details about the programs. Those who could recall why past interventions did not work gave the following reasons: “inability to engage the teenagers’ attention, being too short in duration, or being inappropriate to the cultural context.”

**Table 3:** Perceptions on prevention programs.

Has there been any teen pregnancy prevention programs in Cotacachi?				
	Yes	No	I do not know	No response given
Teenagers	20.8%	29.2%	50%	0%
Adults key informants	33.3%	66.7%	0%	0%
Do you think that a program that combines economic empowerment with sexual health education could decrease the rate of teenage pregnancy in Cotacachi?				
	Yes	No	Maybe	I do not know
Teenagers	45.8%	8.3%	37.5%	8.3%
Adults key informants	80%	0%	20%	0%

#### 3.3.2 Multifaceted teenage pregnancy prevention program acceptability

To evaluate this topic, we made the following question: “Do you think that a program that combines economic empowerment with sexual health education could decrease the rate of teenage pregnancy in Cotacachi? Please explain your answer.” We organized findings quantitatively (Table 3) and qualitatively (Table 2).

Teenagers that answered “Yes” considered that this kind of intervention could work because it would increase awareness and knowledge regarding teen pregnancy. Many teenage respondents voiced that comprehensive sexual education would decrease their vulnerability.

*“Education is the basis of any society, and it is necessary for young people to be informed and know the reality of sexual education.”*

Participant #19

*“Yes, since this way they could help us raise better awareness about the problem that occurs when getting pregnant at an early age.”*

*Participant #27*

Key informants added that a teen pregnancy prevention program could improve decision-making, further encourage entrepreneurship, and empower women, including the economic field (thereby promoting their financial independence and security).

*“Yes, because an economic empowerment program can offer economic independence to women and therefore leads to less dependence on their partner. Furthermore, if sexual education courses are given in the workshops, women can empower themselves, increase their self-esteem and believe in themselves.”*

*Participant #14*

Teens and adult key informants who answered “maybe” argue that the intervention’s effectiveness would depend on people’s interests. For example, one of the teenagers said:

*“Maybe [the program would work], because some young people might listen to what they are taught but some may not.”*

Participant #31

Another adolescent claimed that a real change could not be achieved if the family and community does not change their ideology about this topic.

*“Because it depends more on the culture, and information from the parents. In the [indigenous] communities from the age of 14 you are already an adult.”*

Participant #22

*One participant, however, thought that the problem could be that “not all households would be able to attend [the economic empowerment workshops].”*

Participant #7

Finally, only a few adolescents considered that this kind of program would not work or answered, “I do not know.” One participant claimed that it is a shared responsibility. More themes can be found in Table 2.

*“To be a good result, both researchers and the general population must take part.”*

Participant #20

#### 3.3.3 Economic and personal empowerment interests

Participants reported their interest in three areas of economic empowerment: financial skills, life skills and vocational skills training (Table 4). On financial skills the question was “Economic empowerment is key within teen pregnancy prevention programs. The following are some relevant topics when starting a business and, in general, in financial education. Select the ones that you think teenagers between 13 and 19 years old will find useful and interesting (you can choose several options):”

**Table 4:** Economic empowerment, life, and vocational skills topics of interest.

Topics	Teenagers (*n* = 24)	Key informants (*n* = 15)
	Financial	
Accounting skills	62.5%	60%
Ways to save money	62.5%	66.7%
Budget design	58.3%	80%
Investment and compound interest	50%	33.3%
Financial services in Ecuador	33.3%	40%
Debt management	33.3%	33.3%
Credit management	29.2%	46.7%
	Life skills	
Leadership	79.2%	73.3%
Conflicts resolution	66.7%	86.7%
Time management	62.5%	73.3%
Negotiation skills	58.3%	26.7%
	Vocational skills training	
Computing	83.3%	80%
Auto mechanics training	62.5%	60%
Farming	50%	73.3%
Crafts	50%	46.7%
Sewing	41.7%	53.3%
Carpentry	41.7%	40%
Hairdressing	37.5%	46.7%
Jewelry making	29.2%	33.3%

Teenagers and key informants’ preferences in financial, life skills, and vocational topics.

Overall, we observed that teenagers do not have much interest in financial skills, for example 62.5% reported that they would like to learn about accounting and saving skills, and only 58.3% showed interest in learning budget design. Adult key informants selected the same topics, however, most of them choose budget design, in contrast with teenagers’ preferences. We also ask for suggestions that were not included in our list. We identified that both adolescents and adults suggest that entrepreneurship should be included in the prevention program.

On life skills, the question was “What “life skills” tools would be important for the empowerment of an adolescent in Cotacachi? (you can choose several skills).” A list was provided. Leadership was the life skill most chosen by teenagers (79.2%), and conflicts resolution was most chosen by adult key informants (86.7%). Adolescents also suggested that the program should include training in communication skills, teamwork, responsibility, resilience and independence. In addition to this topics, adult key informants suggested: critical thinking, creativity/innovation, rights and obligations, self-esteem, life purposes, decision making, organization and perseverance.

Finally, on vocational skills training, computing was chosen as the skill in which young people from Cotacachi should be trained to generate income (83.3% of teenagers and 80% of adult key informants). The skills that adolescents suggested were gastronomy, art (music, painting, dance), driving, how to access to college, tourism, first aid, bakery, secretary, waiter, and marketing. Adult key informants suggested: clothing manufacturing, pottery, tourism, first aid/health, and leather crafting.

#### 3.3.4 Sexual health education topics of interest

To explore their thoughts on this area, we made the following question: “Which themes do you think should be covered in sexual health education workshops for teenagers?”

Teenagers and adult key informants have different priorities in terms of sexual education for teenagers, those can be seen in Table 5. All adult key informants considered that life goals and family planning should be taught in sexual health education workshops. However, most teens (92%) selected the topic HIV/AIDS prevention, and only 50% of them chose life goals and family planning.

**Table 5:** Sexual education topics of interest.

Topics	Teenagers (*n* = 24)	Adult key informants *n* = (15)
HIV/AIDS prevention	91.7%	40%
Sexually transmitted infections	75%	93.3%
Consent and rape prevention	70.8%	66.7%
Use of contraceptive methods	66.7%	86.7%
How to obtain contraceptive methods?	62.5%	73.3%
Pregnancy	54.2%	80%
Life goals and family planning	50%	100%
Menstruation and related diseases	41.7%	46.7%
Child marriage and domestic violence	41.7%	60%

Family planning and contraceptive methods sometimes are used as interchangeable terms in Ecuador. But, family planning is the action to decide whether to have children or not; and when and how many children a person wants to have. In contrast, contraceptive methods are specific for a person who does not want to have children at that moment or never (depending on the method). We wanted to check if teenagers and adults key informants differentiate these terms.

In addition to the topics proposed, adolescents suggested that the program should include training about how to properly take care of kids, relationships, harassment, sexual orientations, gender-based violence and masturbation. They also suggested that there should be workshops that include parents to “talk about sexuality with their children” Participant #7.

Adult key informants agreed with adolescents on the themes of childcare, and relationships, but they also suggested anatomy and physiology, how to act in a violence case, Human Rights, risks of teenage pregnancy, abortion, and mental health.

## 4 Discussion

We evaluated the perspectives of teenage and adult key informants from Cotacachi, a small city in northern Ecuador, about teenage pregnancy and their acceptability of a program that combines economic empowerment -through vocational and financial training- with sexual health education to prevent teenage pregnancy. Regarding the study subjects’ perceptions of teen pregnancy incidence, most key informants (67%) pointed out that it is very common, meanwhile, most teenagers (75%) thought it was fairly common. These discrepancies (Table 2) in the perceived commonality of teen pregnancy between key informants and teenagers highlight the need for a nuanced understanding. Despite the discrepancies found, both groups concluded that it happens mainly due to lack of comprehensive sexual education and little parental or family support. This reaffirms the need to systematically implement comprehensive sexual education - an urgent need identified in similar teenage perspectives studies (29–31). From a race/ethnicity perspective, most teenagers did not associate higher pregnancy rates with a specific ethnic group. This contrasted with the adult key informants’ view. Most of adult key informants explained that indigenous teenagers are more vulnerable to pregnancy due to poverty, lack of education, cultural customs, and machismo. Despite the teenagers’ perception, teen pregnancy rates are higher among Ecuadorian indigenous youth. For instance, the frequency of teenage motherhood is 18.3% in indigenous teenagers versus 16.8% in non-indigenous teens between 15–19 years old (9). Additionally, indigenous teenagers in Bolivia, Guatemala, Ecuador, and Nicaragua have larger unmet needs for family planning than non-indigenous youth (8).

While exploring past programs’ effectiveness, we found that nearly none of the adolescents were exposed to an intervention that seek to prevent teenage pregnancy (Table 3). The inadequacy of past programs, attributed to issues like short duration and cultural mismatch, highlights the necessity for programs to not only engage teenagers but also prioritize long-term strategies. We also found that there is an imperative need for culturally adapted interventions to tackle teen pregnancy effectively, particularly in highly indigenous settings.

By exploring the perspective of adult key informants and adolescents on a multifaceted program to prevent adolescent pregnancy, the study reveals a positive outlook from both teenagers and adult key informants regarding the potential success of a comprehensive teen pregnancy prevention program. Notably, teenagers emphasize the importance of reliable sexual health knowledge, while adult key informants stress the pivotal role of economic empowerment. Furthermore, it is interesting that adolescents would like their parents and family to be able to contribute to their training in sexual and reproductive health. This can be explained by the fact that the indigenous population is characterized by maintaining community life as one of the pillars of their cosmovision. The convergence of these perspectives emphasizes the holistic nature of successful interventions.

In terms of economic empowerment, we found that teenagers were less interested in financial education topics compared to adult key informants (Table 4). While little more than half of the teenagers chose accounting skills as the most important topic to learn in the financial area, more than three-quarters of adult key informants selected budget design as the most important topic. Future programs should take these differences into account when adapting an intervention directed to teenagers so that the programs would increase the chance of captivating teens’ interests and involve them in the economic empowerment intervention.

Furthermore, we gathered key informants’ and teenagers’ interests in life and vocational skills topics. Both teens and adult key informants have the same top three life and vocational skill preferences: basic computer capabilities, auto mechanics training, and farming (Table 4). Their interests diverge from the courses on income-generating activities developed in other comprehensive teenage prevention programs (17). Understanding teenagers’ preferences in this area is crucial to design highly accepted courses on income-generating activities.

Finally, we found major differences in sexual health education topics of interest between both groups. While most (92%) teenagers prioritized HIV/AIDS prevention to be part of reproductive health workshops, less than half (40%) of adult key informants selected this topic (Table 5). Meanwhile, all adult key informants agreed that life goals and family planning should be taught in sexual and reproductive health workshops aimed at teenagers, but just half of teenagers were interested in this topic. This finding reinforces the idea that sex education must consider young people’s needs since they do indeed differ from those of adults (29).

The study’s strengths lie in its inclusive representation of indigenous participants, offering unique insights into their perspectives on teen pregnancy prevention. The study’s influence on the design of an ongoing intervention, involving teenagers from the outset, exemplifies a community-based participatory approach. However, limitations such as a small sample size and optional questions must be acknowledged, impacting generalizability. Therefore, results should be interpreted with caution. Furthermore, potential biases, including selective memory and social desirability, should be considered when interpreting results.

The study lays the groundwork for future research, emphasizing the need for larger, more diverse samples to enhance generalizability. The identified preferences and disparities among key informants and teenagers provide valuable guidance for tailoring future interventions. The ongoing digital intervention, with the involvement of a medical doctor from the indigenous population, exemplifies a promising approach to bridge the knowledge-action gap, ensuring cultural sensitivity in program implementation. Continued research is essential to ascertain the viability of teen pregnancy prevention programs in rural and indigenous settings, addressing the unique challenges and opportunities presented by these contexts.

## Data availability statement

The original contributions presented in the study are included in the article/supplementary materials, further inquiries can be directed to the corresponding author.

## Ethics statement

The studies involving humans were approved by Comité de Ética de Investigación en Seres Humanos (CEISH) de la Universidad San Francisco de Quito. The studies were conducted in accordance with the local legislation and institutional requirements. Written informed consent for participation in this study was provided by the participants’ legal guardians/next of kin.

## Author contributions

AT: Data curation, Formal analysis, Investigation, Methodology, Supervision, Writing – original draft, Writing – review & editing. KH: Conceptualization, Formal analysis, Investigation, Methodology, Writing – original draft, Writing – review & editing. OG: Conceptualization, Formal analysis, Investigation, Methodology, Supervision, Writing – review & editing. IP: Conceptualization, Formal analysis, Investigation, Methodology, Supervision, Writing – review & editing.

## Footnotes

^1^Mestizo in Ecuador in defined as “The result of crossing white with Indian.” Arteaga, M. D. Los hijos y las hijas de África y sus descendientes en Cuenca de las Indias durante el gobierno de los Habsburgo 2014;(65):296.
